# PTEN is a negative regulator of mitotic checkpoint complex during the cell cycle

**DOI:** 10.1186/s40164-017-0079-0

**Published:** 2017-06-29

**Authors:** Byeong H. Choi, Steve Xie, Wei Dai

**Affiliations:** 1 Departments of Environmental Medicine, Biochemistry and Molecular Pharmacology, New York University Langone Medical Center, Tuxedo, NY 10987 USA; 2 Institute of Pathology, Kings County Hospital Center, Brooklyn, NY USA; 3 Department of Environmental Medicine, New York University School of Medicine, 57 Old Forge Road, Tuxedo, NY 10987 USA

**Keywords:** PTEN, Mitosis, Mitotic checkpoint complex, Nuclear localization, Chromosomes

## Abstract

Nuclear PTEN plays an important role during mitosis. To understand the molecular basis by which PTEN mediates mitotic progression, we examined whether PTEN regulated the formation of mitotic checkpoint complex (MCC). We observed that arsenic trioxide, a mitotic inducer, stimulated nuclear translocation of PTEN in a time-dependent manner. PTEN physically interacted with Cdc20 and Mad2, two important components of MCC. Arsenic treatment diminished the physical association of PTEN with BubR1 and Bub3 but not with Cdc20 and Mad2. Our further studies revealed that downregulation of PTEN via RNAi enhanced formation of MCC during the cell cycle. Moreover, PTEN silencing induced chromosomal instability. Given the crucial role of PTEN in suppressing tumor development, our study strongly suggests that PTEN also functions to maintain chromosomal stability, partly through suppressing unscheduled formation of MCC.

## Background

Arsenic is a “double-edged” sword. Arsenic trioxide (As_2_O_3_) is effective in the treatment of relapsed acute promyelocytic leukemia [1–3]. On the other hand, arsenic is also an environmental carcinogen [4, 5]. Chronic exposure to this metalloid can induce tumor development in several organs [6–8]. At the cellular level, arsenic treatment induces mitotic arrest [9–11]. Although its molecular basis of mitotic induction remains elusive the mitotic arrest induced by arsenic can be partly due to the perturbation of microtubules and slowing down the cell cycle [12–15].

Phosphatase and tensin homolog (PTEN) is a well-known tumor suppressor as it is mutated at a high frequency in a variety of human malignancies, and inherited PTEN mutations cause cancer-susceptibility conditions [16–19]. Biochemically, PTEN dephosphorylates the lipid second messenger phosphatidylinositol 3,4,5-trisphosphate to generate phosphatidylinositol 3,4-bisphosphate and, by doing so, antagonizes the PI3K/Akt signaling pathway. The PTEN level and its activity profoundly influence cell growth, survival, and tumor susceptibility [20, 21]. A number of studies in the past a few years show that PTEN also has nuclear functions [22, 23]. PTEN plays a role in the maintenance of chromosomal stability through the physical interaction with centromeres and control of DNA repair [24] and nuclear PTEN directly enhances the activity of APC/C by promoting its association with CDH1 [25].

During mitosis, a protein entity termed mitotic checkpoint complex (MCC) is detectable. MCC consists of core spindle checkpoint proteins including Cdc20, Mad2, BubR1, and Bub3 [26]. MCC can be assembled during mitosis without kinetochore localization. We have previously shown that PTEN accumulates in the nucleus during mitosis [27, 28]. Given that molecular regulation of nuclear PTEN and its exact function remain poorly understood, we asked whether PTEN might be involved in regulating the formation of MCC in mitosis, thus affecting the checkpoint function. We observed that treatment with arsenic trioxide induced nuclear translocation of PTEN. PTEN was bound to Cdc20 and Mad2, especially after arsenic treatment. However, arsenic exposure greatly suppressed the formation of MCC. RNAi-mediated downregulation of PTEN promoted the formation of MCC in the presence of arsenic. Given that PTEN silencing induced chromosomal instability, our combined studies suggest that PTEN may regulate the spindle checkpoint through disassembly of MCC during mitotic progression.

## Methods

### Cell culture

HEK293T and HeLa cell lines obtained from the American Type Culture Collection were cultured in DMEM supplemented with 10% fetal bovine serum (FBS, Invitogen) and antibiotics (100 μg/ml of penicillin and 50 μg/ml of streptomycin sulfate, Invitrogen) at 37 °C under 5% CO_2_.

### Antibodies and reagents

Antibodies to PTEN, PARP, Flag and α-Tubulin were purchased from Cell Signaling Technology Inc. Rabbit polyclonal antibodies for BubR1 was developed in the laboratory. Bub3 and Cdc20 antibodies were purchased from Santa Cruz Biotechnology. Human IgGs against centromere proteins (CREST) were purchased from Antibodies Inc. Arsenic trioxide was purchased from Sigma Aldrich.

### Plasmids and siRNA transfection

Flag-tagged Mad2 and Cdc20 were obtained from Addgene. Plasmids were transfected into HEK293T with either LF2000 (Invitrogen) following the manufacturers’ protocol. Transfection efficiency was estimated to be between 80 and 100% in all cases through transfecting a GFP expressing plasmid (data not shown). Human ON-TARGETplus SMARTpool siRNA oligonucleotides that specifically target PTEN (L-003023-00-0010) were purchased from Dharmacon. Individual sets of siRNAs were transfected into HeLa cells with Dharmafect I according to the protocol provided by the supplier. Briefly, cells seeded at 50% confluence in an antibiotic-free culture medium were transfected with siRNA duplexes at a final concentration of 100 nM for 24 h. Small interfering RNAs targeting firefly (*Photinus pyralis*) luciferase (5′-UUCCTACGCTGAGTACTTCGA-3′, GL-3 from Dharmacon) were used as negative control for transfection.

### Cell cycle synchronization

HEK293T cells were synchronized at the G1/S boundary by double-thymidine blocks. Briefly, cells were treated with 2 mM thymidine for 18 h followed by a 9 h release; these cells were treated with 2 mM thymidine for another 18 h and then released into the cell cycle for various times.

### Preparation of protein extracts and immunoblotting

Total cell lysates were prepared in a buffer (50 mM Tris–HCl (pH 7.5), 150 mM NaCl, 1% IGEPAL, 0.1% SDS, and 0.5% sodium deoxycholate) supplemented with a mixture of protease and phosphatase inhibitors. Chromatin and cytosolic/soluble extracts were obtained as described previously [28]. In brief, cell extracts were prepared in the harvest buffer (10 mM HEPES (pH 8.0), 50 mM NaCl, 0.5 M sucrose, 0.1 M EDTA, 0.5% Triton X-100) containing both protease inhibitors (1 mM dithiothreitol (DTT), 2 mg/ml pepstatin, 4 mg/ml aprotinin, 100 mM PMSF) and phosphatase inhibitors (10 mM tetrasodium pyrophosphate, 100 mM NaF, 17.5 mM β-glycerophosphate). The low speed supernatant (500 × g) containing cytoplasmic proteins was collected, and nuclear extracts were made by vortexing the nuclei for 15 min at 4 °C in a buffer containing 20 mM HEPES (pH 7.9), 400 mM NaCl, 1 mM EDTA, 1 mM EGTA, 0.1% IGEPAL CA-630, and protease inhibitors. Protein concentrations were measured using the bicinchoninic acid protein assay reagent kit (Pierce Chemical Co). Equal amounts of protein lysates from various samples were used for SDS-PAGE analysis followed by immunoblotting. Specific signals on immunoblots (polyvinylidene difluoride) were visualized using enhanced chemiluminescence (Super-Signal; Pierce Chemical Co.).

### Immunoprecipitation

Cells were lysed in TBSN buffer [20 mM Tris–Cl (pH 8.0), 150 mM NaCl, 0.5% NP-40, 5 mM EGTA, 1.5 mM EDTA, 0.5 mM Na_3_VO_4_, and 20 mM β-Glycerol phosphate]. The cell lysates were clarified by centrifugation at 15,000×*g* for 20 min at 4 °C. Cleared lysates (1 mg) were added to Flag M2 agarose (Sigma) followed by incubation in the TBSN buffer for 1 h at 4 °C. After incubation, proteins bound to each resin were washed extensively with the binding buffer, eluted in the SDS-PAGE sample buffer, and analyzed by SDS-PAGE.

### Immunofluorescence microscopy

Fluorescence microscopy was performed essentially as described [29]. Briefly, cells were fixed with 4% paraformaldehyde (w/v) for 30 min, washed 3 times in PBS-T (PBS-Tween 0.1%), and permeabilized with 0.1% Triton X-100 in PBS before blocking with 4% Bovine serum albumin (BSA) in PBS-T for 30 min. Primary antibodies were incubated for 1 h, and secondary anti-bodies conjugated to Alexa-Fluor 488 and 555 were incubated for 30 min at room temperature in 4% BSA. DAPI staining was per-formed for 10 min in PBS. After washing, coverslips were dried and mounted on glass slides. Fluorescence microscopy was per-formed using a Leica Confocal Laser Scanning Microscopes SP2 and SL2 using the 100× objective (1.4).

### Statistical analysis

Each experiment was performed at least 3 times. The data were plotted as the mean ± SD. Student t test was used for all comparisons. A P value of <0.05 was considered statistically significant.

## Results

**Fig. 1:**
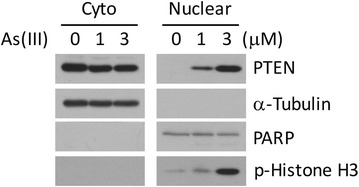
Nuclear translocation of PTEN after arsenic treatment. HeLa cells were treated with arsenic trioxide [As(III)] for various concentrations as indicated, after which cells were collected for obtaining cytoplasmic and nuclear fractions. Equal amounts of cytoplasmic and nuclear proteins were blotted for PTEN, tubulin, PARP, and phospho-histone H3

Given our previous observations that nuclear PTEN plays an important role in mediating mitotic progression [27, 28], we asked whether arsenic affected PTEN nuclear localization. We observed that arsenic treatment induced nuclear translocation in a concentration-dependent manner (Fig. 1). Arsenic treatment also induced significant mitotic arrest as phosphorylated histone H3 also was elevated in which paralleled the increase of nuclear PTEN. Arsenic did not induce significant apoptosis as revealed by blotting with anti-body to PARP-1.

**Fig. 2:**
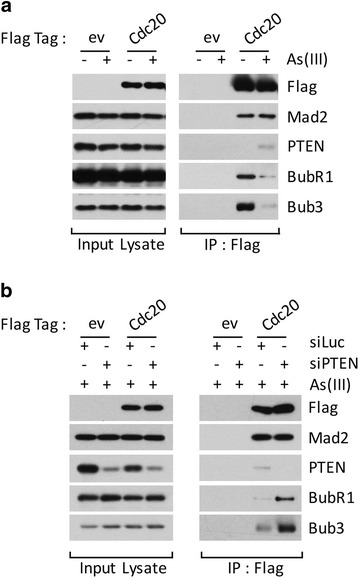
PTEN negatively regulates formation of mitotic checkpoint complex. **a** HEK293 cells were transfected with Flag-Cdc20 expression plasmid or empty vector (ev) for 24 h and then treated with arsenic (1 μM) for 18 h. Equal amounts cell lysates were immunoprecipitated with the anti-Flag antibody. Flag immunoprecipitates, along with lysate inputs, were blotted for Flag, Mad2, PTEN, BubR1 and Bub3. **b** HEK293 cells were co-transfected with Flag-Cdc20 and/or PTEN siRNAs, or control siRNAs (siRNAs to luciferase), for 24 h and treated with arsenic for 18 h. Equal amounts of cell lysates were then immunoprecipitated with the anti-Flag antibody. Flag immunoprecipitates, along with lysate inputs, were blotted for Flag, Mad2, PTEN, BubR1 and Bub3

As mitotic checkpoint complex (MCC) plays an important role in regulating mitotic progression, we asked whether PTEN was involved in physical and/or functional interaction with MCC. Cells transfected with Flag-Cdc20 or vector alone were treated with or without arsenic trioxide. Equal amounts of cell lysates were then immunoprecipitated with the anti-Flag antibody. We observed that Cdc20 interacted with PTEN only after arsenic treatment (Fig. 2a). Significantly, Cdc20’s association with PTEN was inversely correlated with its interaction with BubR1 and Bub3, two major MCC components [30]. On the other hand, Cdc20 interacted with Mad2 efficiently regardless the presence or absence of arsenic.

To determine whether PTEN played a negative role in the formation of MCC, we down-regulated PTEN expression via RNAi approach. We observed that down-regulation of endogenous PTEN led to enhanced association of BubR1 and Bub3 with Cdc20 in the presence of arsenic trioxide (Fig. 2b), strongly suggesting that PTEN is negatively involved in the assembly of MCC in cells exposed to arsenic. Again, the interaction between Cdc20 and Mad2 was not affected by arsenic treatment.

**Fig. 3:**
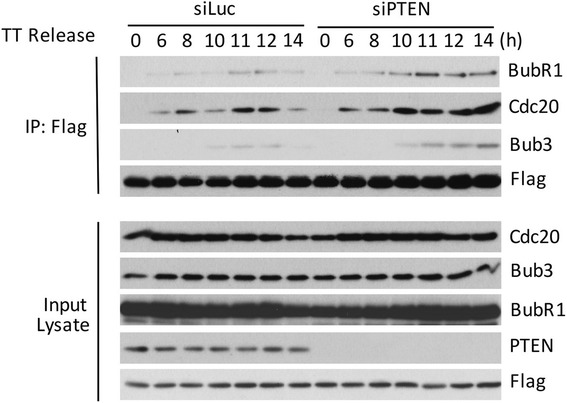
PTEN suppresses MCC formation during the cell cycle. HeLa cells co-transfected with siRNAs to PTEN (siPTEN), or to luciferase (siLuc) as control, and Flag-Mad2 expression plasmid were synchronized at G1/S junction via double-thymidine treatment. At various times after releasing into the cell cycle, cells were collected and lysed. Equal amounts of cell lysates were immunoprecipitated with the anti-Flag antibody. Flag immunoprecipitates, along with lysate inputs, were blotted for Flag, PTEN, Cdc20, BubR1 and Bub3. *TT release* double thymidine release

To further determine whether PTEN exerted a negate effect on the formation of MCC, we first co-transfected cells with Flag-Mad2 expression plasmid and siRNAs to PTEN (or to luciferase as control) for 24 h before starting cell cycle synchronization with double-thymine treatment [31, 32]. Cells released into the cell cycle were subjected to pull-down analysis with the anti-Flag antibody. We observed that transfection of PTEN siRNAs promoted association of BubR1, Bub 3, and Cdc20 with Flag-Mad2, strongly suggesting that PTEN plays a negatively role in the assembly of MCC during mitosis (Fig. 3). PTEN expression was efficiently silenced after transfection of specific siRNAs, which did not significantly impact on expression of Cdc20, Bub3, BubR1 and transfected Flag-Mad2.

**Fig. 4:**
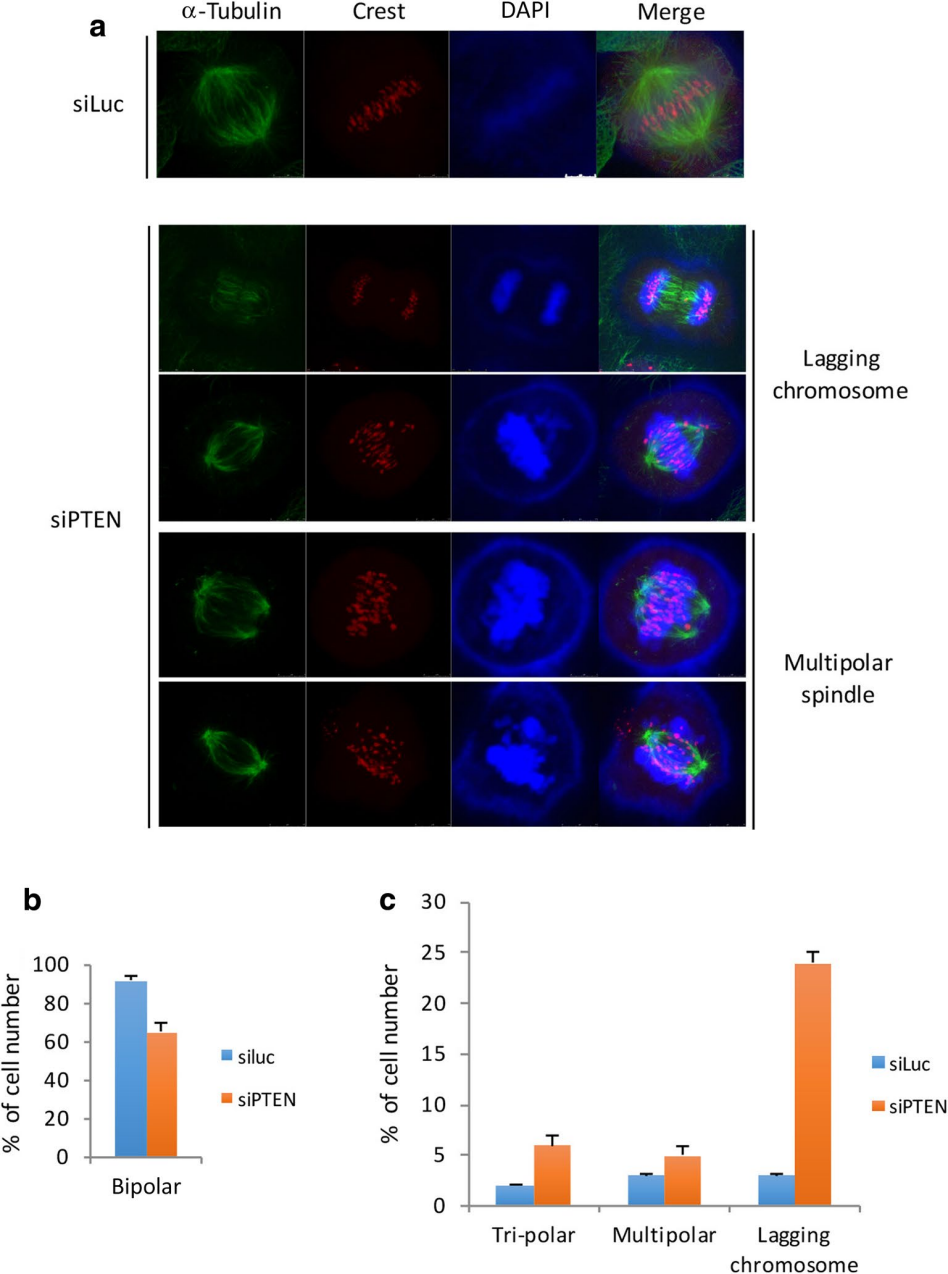
PTEN silencing induces chromosomal instability. **a** HeLa cells seeded in chamber slides were transfected with siRNAs to PTEN or luciferase for 48 h, after which cells were fixed and stained with antibodies to tubulin and CREST. DAPI was also used to label DNA. Stained cells were examined under a fluorescent microscope and representative images are shown. **b**, **c** Percentages of normal or abnormal mitotic cells as shown in **a** were summarized and presented

Spindle checkpoint components including Mad2 and BubR1 function to monitor chromosome congregation during early mitosis and chromosome segregation at the metaphase-anaphase junction [33, 34]. We next examined whether silencing of PTEN had a direct effect on chromosome segregation given the observed physical association of PTEN with Mad2 and Cdc20. We observed that transfection of PTEN siRNAs caused a significant increase in lagging chromosomes and formation of abnormal (tri- and multi-polar) spindle poles with a concomitant decrease in bipolar spindles (Fig. 4).

## Discussion

Although As(III) is recognized as a carcinogen it remains unclear how arsenic perturbs cellular and molecular processes, leading to malignant transformation. It is well documented that As(III) induces major cytogenetic changes, including the formation of micronuclei, chromosomal aberrations, and aneuploidy [36–38]. In fact, nuclear abnormalities are reliable biomarkers for detecting arsenic exposure and its toxicity [36–37]. Well-controlled studies in rodent cells also demonstrate that treatment with As(III) significantly increased the number of aberrant chromosomes and the frequency of micronuclei, which is coupled with an alteration of mitotic index [39]. Thus, cytogenetic alterations and chromosome instability (CIN) induced by arsenic compounds may play a causative role in genomic instability, malignant transformation, and cancer development [40]. In the current study, we have demonstrated that As(III) treatment has a significant effect on PTEN subcellular localization and its function. The observation that arsenic induces PTEN nuclear accumulation is likely due to its effect on mitotic arrest [10, 11], which is also supported by enhanced levels of phospho-H3 (Fig. 1). As reported in our early studies, PTEN undergoes cell-cycle dependent nuclear localization, peaking at mitosis [27, 28]. It is conceivable that deregulated PTEN regulation due to exposure to As(III) would exert a negative impact on mitotic progression, leading to CIN and aneuploidy.

Deregulated PTEN functions are frequently observed in human cancers that exhibit increased genomic instability and aneuploidy [41, 42]. We and others have observed PTEN localization at mitotic apparatuses including centrosomes and centromeres/kinetochores in mitotic cells [24, 27, 43]. PTEN and the PI3K signaling pathway appear to play a role in centrosome composition and integrity [43], which are essential for faithful segregation of chromosomes during cell division. It has been shown that PTEN loss partially compromises cell cycle checkpoint, leading to enhanced genomic instability [44, 45], which is mediated partially through regulating expression of Rad51, a component involved in DNA double-strand repair and homologous recombination [24]. Consistent with this observation, we have shown that PTEN interacts with Rad52 and regulates its activity [29]. In addition, PTEN is modified by sumoylation [46], which controls its nuclear localization and responses to genotoxic stress [47]. In the current study, we observe that PTEN is physically associated with Mad2 and Cdc20 and that PTEN disrupts the interaction of these two checkpoint proteins with other MCC components (BubR1 and Bub3). These observations suggest that PTEN may function as a negative regulator of MCC during mitotic progression. The spindle checkpoint is tightly regulated and overexpression of checkpoint components may also cause chromosomal instability, aneuploidy and tumor development [19, 33, 34]. Therefore, it is conceivable that MCC needs to be removed in a timely manner during mitotic progression and that PTEN may play an essential role in this process.

Based on our current and previous studies, we propose a simple model that explain the mode of action of PTEN. PTEN protein undergoes cell cycle-dependent nuclear translocation, peaking in mitosis. Nuclear PTEN physically interacts with Cdc20 and/or Mad2 and their interaction has a direct impact on the assembly or disassembly of MCC, thus regulating mitotic progression. Deregulated mitosis frequently leads to chromosomal instability.
